# Ultrasound scanning by emergency physicians in jaundice patient

**DOI:** 10.1186/2036-7902-7-S1-A18

**Published:** 2015-03-09

**Authors:** M Algaba-Montes, A Oviedo-García, J Lopez-Libano, JM Alvarez-Franco, A Segura Grau

**Affiliations:** 1 Emergency Department, Valme Hospital, Seville, Spain; 2 Critical Care Department, Miramar Hospital, Mallorca, Member of the Working Group of ultrasound SEMERGEN, Spain; 3 Emergency Department, IB-Salut, Ibiza, Member of the Working Group of ultrasound SEMERGEN, Spain; 4 MFYC. Médico ecografista en Centro Diagnostico Ecográfico y en Unidad de ecografía general del Hospital San Francisco de Asis, Madrid Spain

## Background

Abdominal ultrasound has proven to be a useful, safe, versatile, with appropriate experience, help earlier diagnosis and comprehensive management of patients seen in the emergency department.

## Objective

We present a case of Jaundice by biliary stent obstruction in patient with pancreatic neoplasia, through a bedside ultrasound by Emergency Physicians.

## Patients and methods

We report the case of a patient of 64 years old with pancreatic neoplasia bearer of 8 French plastic stent by ERCP for biliary drainage for 6 days as palliative treatment, admitted in ER by jaundice and abdominal pain in epigastrium.

## Results

On arrival had malaise, was hypotensive, febrile, tachycardic and universal jaundice. The analyzes highlighted a bilirubin of 9.52 at the expense of direct fraction (8.60 mg / dl), and 22.500 leukocytes with neutrophilia. She underwent a bedside abdominal ultrasound where we found a central hyperechoic endoprosthetic image with acoustic shadowing, compatible with biliary litiasis. The patient was derived for urgent endoscopic retrograde cholangiopancreatography, sphincterotomy, removal of prosthesis, washing and placement 10 French prostheses, allowing a favorable evolution of the patient discharged within 72 hours without complications.

## Conclusion

The use of stents as temporary drainage of the bile duct as palliative treatment for pancreatic head tumors is an effective, useful, safe and effective, it also represents a decrease in short-term mortality in these patients, and improved survival. The use of ultrasound in emergency allows us greater agility and speed in the diagnosis of prosthetic obstructions, allowing a more integrated management of the same. As shown in the case that concerns us a bedside ultrasound by Emergency Physicians favored a quick and agile diagnosis of biliary sepsis patient suffering, allowing prompt treatment and an early solution to the problem.

## Informed consent

The study was conducted in accordance with the ethical standards dictated by applicable law. Informed consent was obtained from each owner to enrolment in the study and to the inclusion in this article of information that could potentially lead to their identification.
